# Some Amusing Theories of By-gone Days

**Published:** 1890-04

**Authors:** E. Wildeman

**Affiliations:** Yardley, Pa.


For Daniel's Texas Medical Journal.

### SOME AMUSING THEORIES OF BY-GONE DAYS.

DR. E. WILDEMAN, YARDLEY, PA.

I HAVE been glancing over Martin's Philosophy, which gave the scientific theories of 1778, and concluded the readers of your journal might find some very interesting,—amongst which are:

"The learned of late have found by their Microscopes that not only Man, but all Animals do really exist (in their proper Form compleat in all their parts) in the Seed of the Male Animal, before Generation, in a small invisible State, called Miniature. It is amazing to see the prodigious Numbers of little Creatures, like so many Tadpoles, swimming every way in the Male Sperm of all Animals. Those animals are so small that 3,000,000,000, i. e. three thousand Millions of them, are not equal to a Grain of sand, whose diameter is but the 100dth part of an inch.

"The Animalcule that has the good Luck to get safe into the womb, through the Fallopian tubes is a kind of Egg, is there fostered a while somehow. At length, the Placenta appears like a cloud on one side of the external coat of the egg, and at the same time the Spine of the Embryo is grown so big as to be visible, and a little after the Brain and cerebellum appear like two small Bladders, and the Eyes next stand goggling out of the head. Then the beating of the heart or Punctum Saliens, is plainly to be seen, and the Extremities of the Body discover themselves last of all. So far Dr. Keill. And this is the present Theory of Generation; and very much alike in Plants and Animals.*

*The affair of Impregnation and Generation even after all the late Discoveries, remains so very perplexed and doubtful that nothing of Certainty can yet be determined about it. Some asserting the little "Animal" to be originally in the Female Egg, others deny that, and affirm they have no being till the Egg is fecundated by the Semen Masculinum in Coitu. See the controversy in Mr. Bradley's Phil. Account of the Works of Nature, chap. 9, Miscellanea Curiosa, Vol. 1, page 142, by Mr. Garden.

This is the scientific idea on Generation of 1778.

FOWLS, NATURE OF.—Egg is a stupendous contrivance of infinite wisdom; chickens are nourished by the white alone till grown great, then fed on the stronger diet of the Yolk. Though all fowl are hatched from Eggs, yet it is not always by the incubation or brooding of the parent Fowl but by some other heat or warmth sometimes. Thus Job *** ** The Ostrich leaveth her Eggs in the Earth and Warmeth them in the Dust; and at this Time they hatch Chickens in Ovens at Grand Cairo in Egypt. Each Oven containing 8000 Eggs."

THE LUNGS * * * * * THEIR USE.—"This is the great organ of Breathing or Respiration as the Air by its weight forceth into evry cavity, so as soon as the Fœtus is born It rushes into the cavity of the Lungs and fills the vesicles, and by extending them, compresses the small Blood Globules in the Vessels spread upon them; this compression is much greatest when the air is expelled out of the Lungs by the Contraction of the Breast, and by this compression the Red Globules of Blood which through the languid motion in the veins were grown too large to circulate, are again broken and divided into the Serum, and so the blood is anew made fit for nutrition and secretion. Dr. Keill also thinks that that the air doth hereby enter and mix with the blood. Dr. Cheyne saith, the Elastic Globules of the blood are hereby formed—and others hold other opinions. But I (Martin) think it strange that Etmuller, amongst its fourteen uses thereof, has not mentioned the Vital Spirit of the Air, which, probably, intermixed with the Blood, and diffused through all the Body, and is therein the Principal of Animal Life.—Since it is well known Animals cannot live in Air deprived of this *Spirit*. Neither has Mr. Derham hinted anything concerning it."

Martin's old philosophy continues, describing the Heart, Liver (to separate Bile from the Blood). I notice there is no description of the Spleen.

A DESCRIPTION OF THE CUTICULAR GLANDS is very elaborate, one grain of sand to cover 125,000 of them, etc. But in a note below Martin says: "This is upon trust of Mr. Lewenhoek, who certainly was very happy at these kinds of inventions, for his eyes, though old, and microscopes, though single ones, could in many cases discover what I never could with young eyes and the best *double* microscopes. I have tried in variety of subjects to find these Pores in the Cuticle, *but in vain.* And Indeed I very much question whether he ever did actually see any such Thing. M."

There are many sentences that show to us how our forefathers handled scientific questions. How ambitious they were, and what little hesitation was used to contradict a fellow-scientist's views. You notice it is done in plain English.

When we gaze into these old works, and think how suffering humanity was treated in those days, it gives to us a feeling of increased pride and thankfulness, to know that scientific knowledge has so advanced as to brighten the darkness, and bestow upon us a high grade of intellectual power which can be constantly improved by our text books and medical journals.

